# Shared Clavulanate and Tazobactam Antigenic Determinants Activate T-Cells from Hypersensitive Patients

**DOI:** 10.1021/acs.chemrestox.2c00231

**Published:** 2022-09-22

**Authors:** Adriana Ariza, Kanoot Jaruthamsophon, Xiaoli Meng, Marina Labella, Kareena Adair, Arun Tailor, Chonlaphat Sukasem, Paul Whitaker, Daniel Peckham, Munir Pirmohamed, María José Torres, Dean John Naisbitt

**Affiliations:** † Allergy Research Group, Instituto de Investigación Biomédica de Málaga-IBIMA, 29009 Málaga, Spain; ‡ Department of Pharmacology and Therapeutics, Institute of Systems, Molecular, and Integrative Biology, University of Liverpool, Liverpool L69 3GE, U.K.; § Division of Pharmacogenomics and Personalized Medicine, Department of Pathology, Faculty of Medicine Ramathibodi Hospital, Mahidol University, Bangkok 10400, Thailand; ∥ Allergy Unit, Hospital Regional Universitario de Málaga, 29009 Málaga, Spain; ⊥ Bradford Teaching Hospitals NHS Trust, Bradford BD9 6DA, U.K.; # Regional Adult Cystic Fibrosis Unit, St James’s University Hospital, Leeds LS9 7TF, U.K.; ∇ Andalusian Center for Nanomedicine and Biotechnology-BIONAND, 29590 Málaga, Spain; ○ Departamento de Medicina, Universidad de Málaga, 29071 Málaga, Spain

## Abstract

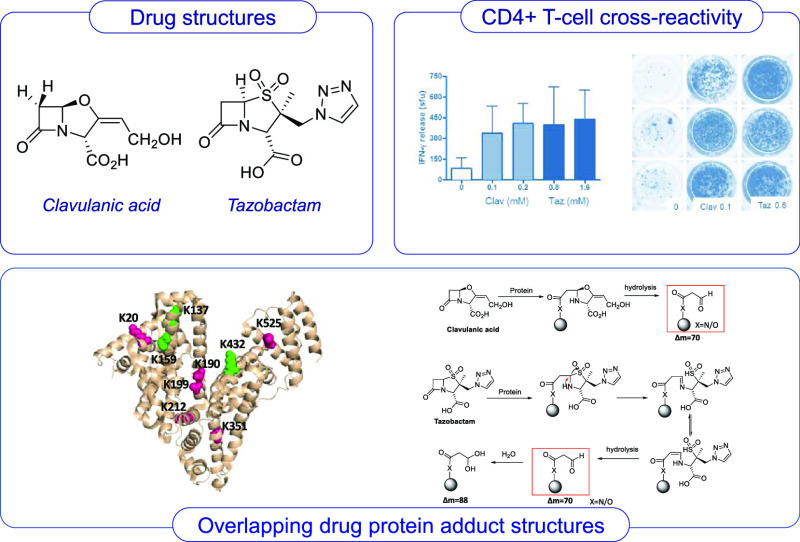

β-Lactamase inhibitors such as clavulanic acid and tazobactam were developed to overcome β-lactam antibiotic resistance. Hypersensitivity reactions to these drugs have not been studied in detail, and the antigenic determinants that activate T-cells have not been defined. The objectives of this study were to (i) characterize clavulanate- and tazobactam-responsive T-cells from hypersensitive patients, (ii) explore clavulanate and tazobactam T-cell crossreactivity, and (iii) define the antigenic determinants that contribute to T-cell reactivity. Antigen specificity, pathways of T-cell activation, and crossreactivity with clavulanate- and tazobactam-specific T-cell clones were assessed by proliferation and cytokine release assays. Antigenic determinants were analyzed by mass spectrometry-based proteomics methods. Clavulanate- and tazobactam-responsive CD4^+^ T-cell clones were stimulated to proliferate and secrete IFN-γ in an MHC class II-restricted and dose-dependent manner. T-cell activation with clavulanate- and tazobactam was dependent on antigen presenting cells because their fixation prevented the T-cell response. Strong crossreactivity was observed between clavulanate- and tazobactam-T-cells; however, neither drug activated β-lactam antibiotic-responsive T-cells. Mass spectrometric analysis revealed that both compounds form multiple antigenic determinants with lysine residues on proteins, including an overlapping aldehyde and hydrated aldehyde adduct with mass additions of 70 and 88 Da, respectively. Collectively, these data show that although clavulanate and tazobactam are structurally distinct, the antigenic determinants formed by both drugs overlap, which explains the observed T-cell cross-reactivity.

## Introduction

**Figure 1:**
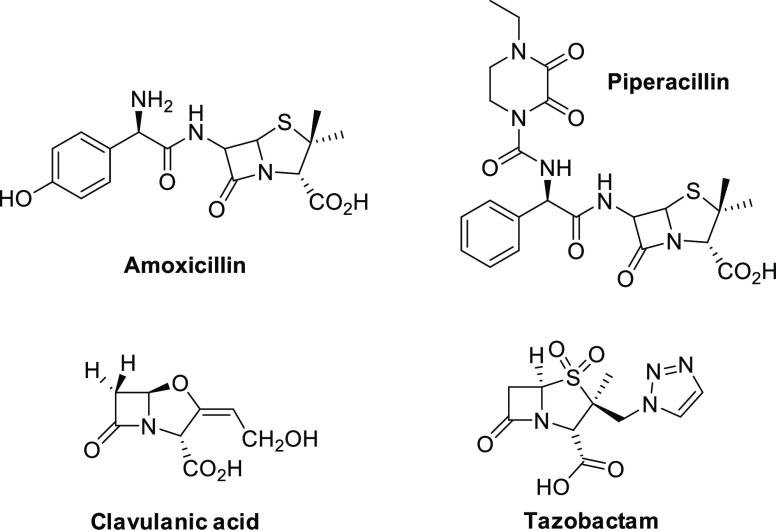
Chemical structure of B-lactam antibiotics and B-lactamase inhibitors.

β-Lactamase inhibitors were developed to overcome increasing β-lactamase-mediated resistance to β-lactam antibiotics.^1^ These structurally distinct drugs (Figure 1) inactivate β-lactamases by irreversibly binding to the active site on the enzyme, thereby allowing β-lactam antibiotics to reach the target site.^2^ Two β-lactamase inhibitors, clavulanic acid and tazobactam, are used in combination with amoxicillin and piperacillin, respectively, significantly improving their clinical efficacy.^3,4^ For example, the addition of clavulanic acid has been shown to significantly increase the activity of amoxicillin against bacteria possessing a β-lactamase such as *Escherichia coli* and *Proteus mirabilis*.^3^ Tazobactam has been shown to increase the activity of piperacillin and lower the minimum inhibitory concentration against a wide variety of pathogens.^5^

Despite showing antimicrobial activity, β-lactam/β-lactamase inhibitor combinations are commonly associated with drug hypersensitivity reactions.^6^ It has been estimated that approximately 10% western population are labeled as β-lactam hypersensitive, although up to 90% of these patients are found not to be truly allergic after clinical evaluation.^7,8^ Extensive studies have demonstrated that β-lactam hypersensitivity is mediated by immunological mechanisms that involve specific IgE antibodies or drug-responsive T-cells. β-Lactam antibiotics form covalent adducts with proteins through opening of the β-lactam ring, and these adducts are recognized by IgE antibodies and stimulate patient T-cells. Recognition of different structural determinants of the β-lactam protein adduct, either the drug side chain, the thiazolidine ring and/or the carrier protein by IgE antibody or T-cells can lead to the drug-specific immune responses.^9^ As expected, there is significant crossreactivity between β-lactam antibiotics, particularly those that share similar side chains.^10^ Although hypersensitivity reactions to β-lactam antibiotics have been extensively studied, the mechanism involved in β-lactamase inhibitor hypersensitivity is less well defined.

Clavulanic acid was initially considered as nonimmunogenic when first marketed in combination with amoxicillin.^11^ However, it has recently been reported to contribute to the hypersensitivity reactions in patients receiving amoxicillin-clavulanic acid.^12−14^ Additionally, selective IgE- and T-cell-mediated reactions to clavulanic acid, with tolerance to amoxicillin, have been reported.^12−17^ Piperacillin-responsive T-cells are commonly detected in piperacillin-tazobactam hypersensitive patients;^18^ however, in rare cases, tazobactam-specific peripheral blood mononuclear cell (PBMC) activation has been detected (unpublished data). Based on the structure of the β-lactamase inhibitors, it is assumed that the risk for crossreactivity to β-lactam antibiotics would be rare.^1^ Indeed, our previous study has demonstrated that T-cells from clavulanic acid hypersensitive patients do not crossreact with amoxicillin,^19^ although simultaneous sensitization to both drugs can exist.^20^ However, the nature of β-lactamase inhibitor antigenic structures and crossreactivity between clavulanic acid and tazobactam has not been evaluated. Thus, this study aimed to characterize clavulanate- and tazobactam-T-cell crossreactivity and examine the antigenic determinants formed by the two drugs.

## Experimental Procedures

### Human Subjects

PBMCs were isolated from three patients with immediate hypersensitivity reactions to clavulanic acid confirmed using the procedure described in the European Network of Drug Allergy,^21,22^ and the patients were recruited to the study for the generation of clavulanate-responsive T-cell clones. Furthermore, tazobactam-responsive T-cell clones were generated from one patient with cystic fibrosis that developed a delayed-type maculopapular eruption following exposure to piperacillin-tazobactam. It would be interesting to speculate that the two study drugs produce these polarized types of reaction; however, a recent study exploring skin and provocation testing in piperacillin-tazobactam hypersensitive patients identified three patients selectively sensitized against tazobactam (displaying cross-reactivity against clavulanic acid) that developed immediate or delayed-type reactions.^23^ The study was conducted according to the Declaration of Helsinki principles and was approved by the local Ethics Committees of Málaga, Spain, and Leeds, UK. All subjects were informed orally about the study, and they signed the corresponding informed consent form.

### Diagnostic Testing

Skin prick tests were performed as recommended and if negative were followed by intradermal tests in patients with immediate clavulanic acid hypersensitivity.^21^ Skin testing is not recommended in the tazobactam hypersensitive patient because of assay insensitivity.^18^ In the skin prick test, a wheal larger than 3 mm with a negative response to the control saline was considered positive. For intradermal testing, the wheal area was marked at the beginning and 20 min after testing. An increase in the wheal diameter greater than 3 mm was considered positive. Venous blood (70 mL) was collected in heparin tubes, and PBMCs were isolated by density gradient separation for lymphocyte transformation testing (LTT) and the generation of drug-responsive T-cell clones.^24^

### Generation of Epstein–Barr Virus (EBV)-Transformed B-Cell Lines

EBV-transformed B-cell lines were generated from PBMCs of the hypersensitive patients by transformation with the supernatant from the EBV-producing cell line B95.8. Autologous (from the same hypersensitive patient) EBV-transformed B-cells were used as a source of antigen presenting cells (APCs) and maintained in RPMI 1640 supplemented with fetal bovine serum (10%, v/v) (Invitrogen, Paisley, UK), l-glutamine (100 mM) penicillin (100 μg/mL), and streptomycin (100 IU/mL) for up to 4 months.

### Medium for T-Cell Culture and Cloning

RPMI 1640 supplemented with pooled heat-inactivated human AB serum (10%, v/v), HEPES (25 mmol/L), l-glutamine (2 mmol/L), transferrin (25 mg/mL), penicillin (100 μg/mL), and streptomycin (100 IU/mL). IL-2 (100 IU/mL) was added when establishing drug-specific T-cell clones. T-cell culture medium without antibiotics was used for testing the generated T-cell clones.

### Generation of T-Cell Clones

PBMCs from clavulanic acid or tazobactam hypersensitive patients (1 × 10^6^ cells/well) were cultured with clavulanate (0.05 and 0.1 mM) or tazobactam (0.1 and 0.25 mM) in T-cell culture medium without antibiotics for 14 days. To maintain antigen specific proliferation, cultures were supplemented with recombinant human IL-2 (200 IU/mL) on days 6 and 9. On day 14, T-cells were serially diluted (0.3–3 cells/well) and subjected to phytohemagglutinin (PHA)-driven expansion (5 mg/mL) using the established methodology.^25,26^

### Specificity of T-Cell Clones

T-cell clones were tested for clavulanate or tazobactam specificity by measuring proliferation. T-cell clones (5 × 10^4^ cells/well) were cultured with autologous irradiated EBV-transformed B cells (1 × 10^4^ cells/well) and clavulanate (0.1 mM) or tazobactam (0.25 mM) for 48 h. Proliferation was measured by the addition of [^3^H]thymidine (0.5 μCi/well, 5 Ci/mmol, Morovek Biochemicals, Brea, CA, USA) for the last 16 h of the culture period. T-cell clones with a stimulation index (SI) greater than 1.5 were selected as potential specific T-cell clones and expanded by repetitive stimulation with irradiated allogenic PBMC (5 × 10^4^ cells/well), PHA (10 μg/mL), and IL-2 (700 IU/mL) for further experiments and specific response confirmation, as described below.

### Proliferative Response and Crossreactivity Studies

Clavulanate- and tazobactam-responsive T-cell clones expanded after initial testing were assayed for dose-dependent proliferative responses with clavulanate (0.025–12.8 mM) and tazobactam (0.025–12.8 mM). Moreover, crossreactivity between both drugs was analyzed. Proliferation was measured by [^3^H]thymidine incorporation, and results were considered positive if the SI was higher than 2.0, as previously described.^27,28^ Amoxicillin- and piperacillin-specific T-cell clones described previously^19,27^ were also expanded to be used in crossreactivity studies with clavulanate and tazobactam.

### Release of IFN-γ by Clavulanate and Tazobactam-Stimulated T-Cell Clones

ELISpot was used to detect IFN-γ released by drug-responsive T-cell clones. T-cell clones (5 × 10^4^ cells/well) were cultured with autologous irradiated EBV-transformed B-cells (1 × 10^4^ cells/well) and clavulanate (0.1 mM) or tazobactam (0.8 mM) on plates precoated with capture antibody for 48 h (Mabtech, Sweden). Plates were then washed, and IFN-γ secreting cells were detected with biotin-conjugated antibodies and streptavidin-HRP, according to the manufacturer’s instructions (Mabtech). Secreting cells were counted using an AID ELIspot reader.

### Phenotype of T-Cell Clones

The CD phenotype of clavulanate- and tazobactam-specific T-cell clones was characterized by flow cytometry. Antibodies used for flow cytometry staining were CD4-fluorescein isothiocyanate (FITC) and CD8-phycoerythrin (PE) (BD, New Jersey, USA).

### Functional Studies of T-Cell Activation

To analyze the mechanistic basis of clavulanate- and tazobactam-specific T-cell activation, CD4^+^ and CD8^+^ T-cell clones were subjected to further experiments to explore the pathways of drug antigen presentation. T-cell clones were cultured with clavulanate or tazobactam in the presence or absence of autologous irradiated APCs or irradiated and fixed APCs (fixation: 0.05% glutaraldehyde for 30 s, 1 mM glycine for 45 s and extensive washing). Furthermore, the importance of MHC molecules in the presentation of clavulanate and tazobactam to specific T-cell clones was tested by pretreating APCs with MHC class-I and II blocking antibodies for 30 min at 37 °C. Activation of T-cells was determined by measurement of proliferation or IFN-γ secretion using [^3^H]thymidine or ELIspot, respectively.

### Concentration-Dependent Modification of Human Serum Albumin (HSA) by Clavulanate or Tazobactam In Vitro

Clavulanate or tazobactam was incubated with HSA (1 mM, 40 μL) in phosphate buffer at 37 °C for 24 h. The molar ratios of drug to protein were 1:1, 5:1, 10:1, 20:1, 100:1, and 200:1. Protein was precipitated twice with nine volumes of ice-cold methanol to remove noncovalently bound drugs, suspended in 50 μL of phosphate buffer, and then reduced with 10 mM dithiothreitol (15 min) and alkylated with 50 mM iodoacetamide (15 min) at room temperature. The protein was precipitated once more with methanol and finally dissolved in 50 μL of 50 mM ammonium bicarbonate, pH 7.0. The protein (400 μg) was incubated with trypsin (2 μg) overnight at 37 °C. Samples of the digest (50 μL) were processed for liquid chromatography–tandem mass spectrometry (LC**–**MS/MS) analysis as described below.

### LC–MS/MS Analysis of Drug Protein Adducts

The tryptic peptides were analyzed using a Triple TOF 6600 mass spectrometer (Sciex). Samples were reconstituted in 50 μL of 0.1% formic acid, and 2 μL of sample was delivered into the instrument using an Eksigent Nano-LC system mounted with a nanoACQUITY UPLC Symmetry C18 Trap Column and an analytical BEH C18 nanoACQUITY Column (Waters, MA, USA). A NanoSpray III source was fitted with a 10 μm inner diameter PicoTip emitter (New Objective). Samples were loaded in 0.1% formic acid onto the trap, which was then washed with 2% ACN/0.1% FA for 10 min at 2 μL/ min before switching in-line with the analytical column. A gradient of 2–50% (v/v) ACN/0.1% (v/v) FA over 90 min was applied to the column at a flow rate of 300 nL/min. Spectra were acquired automatically in positive ion mode using information-dependent acquisition, using mass ranges of 400–1600 Da in MS and 100–1400 amu in MS/MS. Up to 25 MS/MS spectra were acquired per cycle (approximately 10 Hz) using a threshold of 100 counts per s, with dynamic exclusion for 12 s and rolling collision energy.

### Analysis of Drug-Modified Proteins

LC–MS/MS data were searched against the reviewed human proteome (UniProt/SwissProt accessed October 2020), using ProteinPilot software, v4.0 (Sciex). Data were refined using default parameters, and searches were performed with the following parameters: enzymatic cleavage restriction for trypsin, fixed modification-carbamidomethylation of cysteine, variable modifications-methionine oxidation (+15.99), asparagine and glutamine deamidation (+0.98), and drug modification of lysine (+70.1) and (+88.1).

### Statistical Analysis

Quantitative variables are expressed as mean and standard deviation, and comparisons were carried out using the *t* test for mean values.

## Results

### Donor Clinical Characteristics

Patients 1–3 were diagnosed with immediate hypersensitivity reactions to clavulanic acid after the administration of the combination amoxicillin-clavulanic acid (clinical details of reactions in Table 1). Clinical manifestations ranged from urticaria to anaphylactic shock. All patients were skin test positive in immediate reading to clavulanic acid and tolerated the administration of amoxicillin. The tazobactam-exposed patient developed a maculopapular eruption and fever with delayed onset (Table 1). PBMCs from the patient were stimulated to proliferate in the presence of tazobactam (stimulation index 3 or above with 2 tazobactam concentrations), but not piperacillin.

**Table 1:** Clinical Details of Patients with Confirmed Hypersensitivity to Clavulanic Acid or Tazobactam

patient ID	age	gender	drug reaction interval (min)	reaction study interval (days)	reaction	clinical diagnosis
P1	44	F	20	240	anaphylaxis	clavulanic acid hypersensitive (positive intradermal test)
P2	34	F	10	730	urticaria	clavulanic acid hypersensitive (positive intradermal test)
P3	63	M	5	1460	anaphylactic shock	clavulanic acid hypersensitive (positive intradermal test)
P4	34	F	12 (days)	450	maculopapular rash, fever	tazobactam hypersensitive (positive LTT)

### Clavulanate and Tazobactam Stimulate T-Cells from Hypersensitive Patients

**Figure 2:**
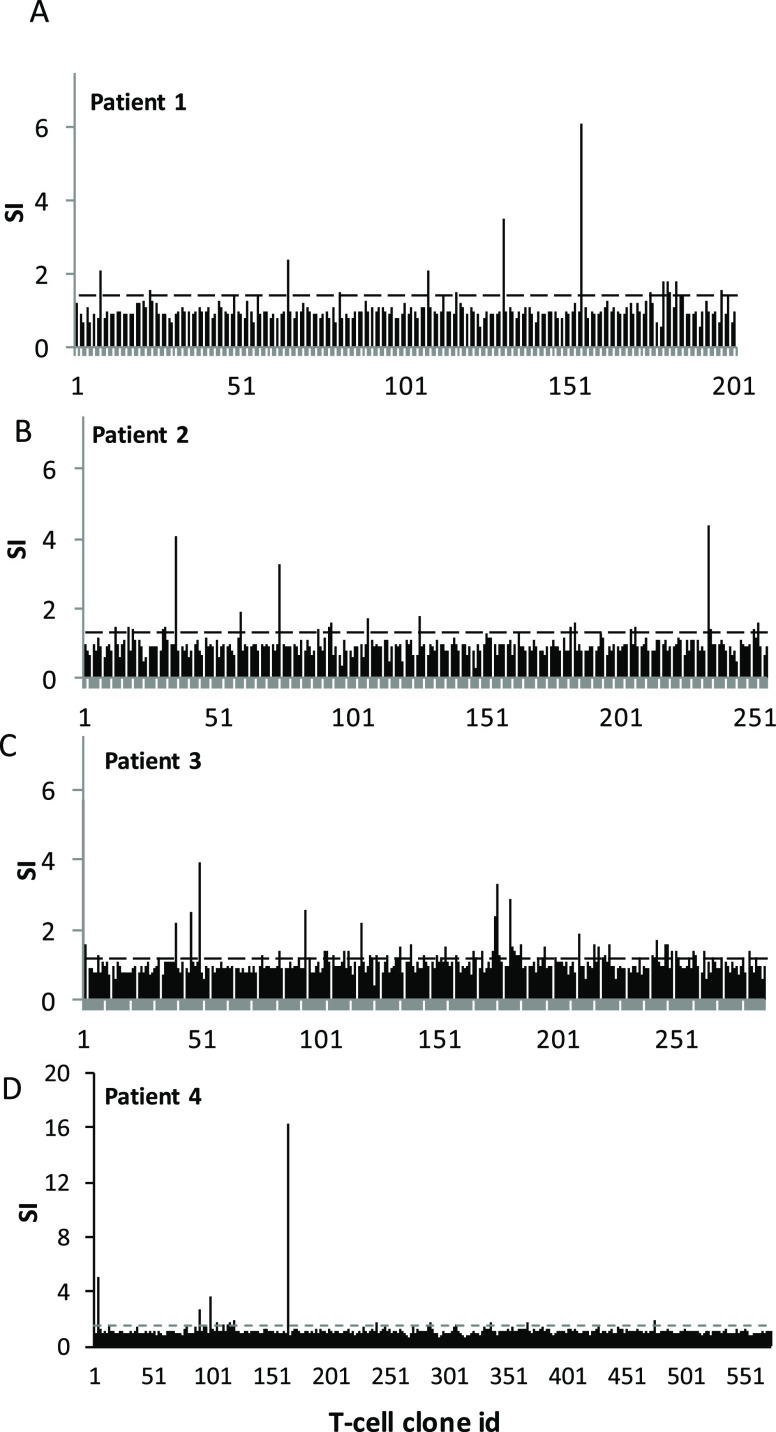
T-cells from clavulanic acid or tazobactam hypersensitivity patients are stimulated by clavulanate or tazobactam. T-cell clones were generated from the blood of clavulanic acid or tazobactam hypersensitive patients. Clones were then cultured with autologous APCs in the presence of medium as a negative control and clavulanate (A–C) or tazobactam (D) for 2 days. Proliferation was measured by the addition of [^3^H]-thymidine. Results are expressed as the SI, calculated by dividing the mean counts per minute (cpm) of drug-stimulated cells by the mean cpm of nonstimulated cells. Clones were expanded for further assessment when SI > 1.5 was recorded.

Clavulanate- treated PBMCs from patients 1–3 and tazobactam-treated PBMCs from patient 4 were serially diluted and subjected to repetitive rounds of stimulation to generate over 1200 T-cell cultures originating from single precursor cells. As described in ref (19), a total of 59 T-cell clones displayed reactivity against clavulanate on initial testing, and these were selected and expanded for additional assessment (Figure 2A–C). A total of 574 clones were generated from tazobactam patient 4 (Figure 2D). Twelve T-cell clones were activated with tazobactam, and these clones were selected for phenotypic assessment and crossreactivity studies.

### Clavulanate- and Tazobactam-Responsive Clones Display Strong Crossreactivity

**Figure 3:**
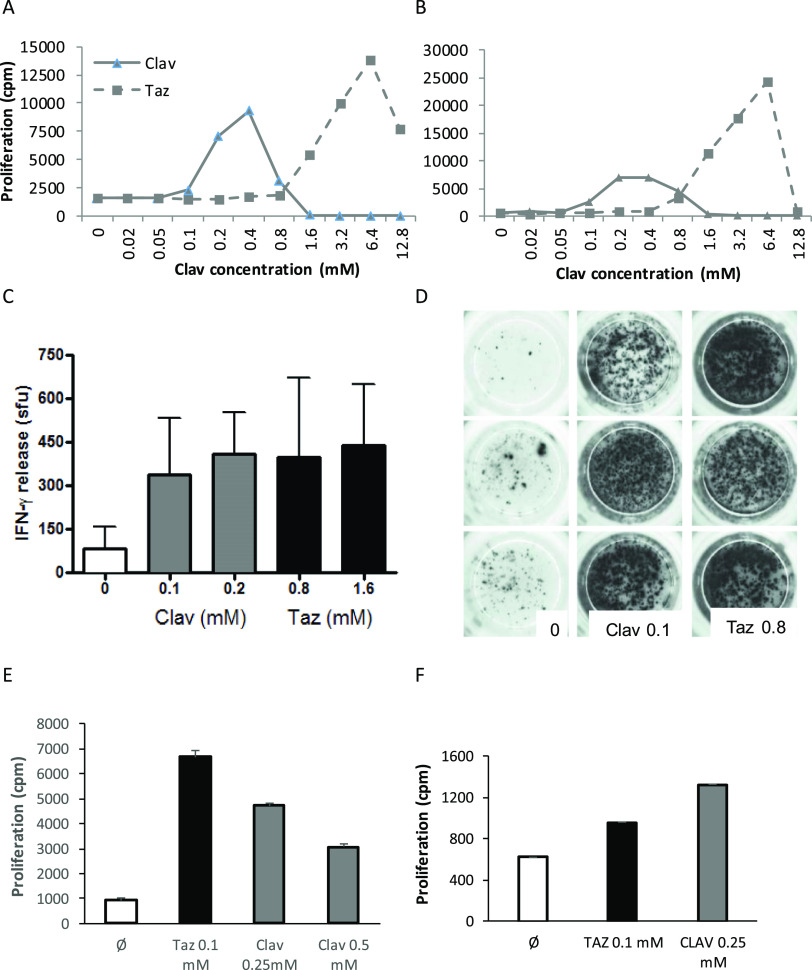
Clavulanate- and tazobactam-responsive T-cell clones are activated with the alternative 6-lactamase inhibitor. (AB) Dose-dependent proliferation of clavulanate T-cell clones with clavulanate and tazobactam. Clones were cultured with autologous APCs in the presence of medium as a negative control and titrated concentrations of the B-lactamase inhibitors for 2 days before the addition of [^3^H]-thymidine. Results are expressed as the mean counts per minute (cpm). (C,D) Clavulanate T-cell clones were cultured with APCs and clavulanate or tazobactam and IFN-P secretion was measured using ELIspot. (E,F) Tazobactam T-cell clones were stimulated to proliferate in the presence of tazobactam and clavulanate. Proliferation was measured by the addition of [^3^H]-thymidine.

T-cell clones were assessed by flow cytometry and found to display the CD4^+^ coreceptor. Clavulanate-responsive T-cell clones were stimulated to proliferate at drug concentrations between 0.05 and 0.8 mM, with higher concentrations associated with drug-induced T-cell toxicity. Crossreactivity with tazobactam was detected with all T-cell clones at tazobactam concentrations up to 6.4 mM (Figure 3A,B). IFN-γ secretion was detected from clavulanate-responsive clones stimulated with both clavulanate and tazobactam (Figure 3C,D). Similarly, the tazobactam-responsive T-cell clones were stimulated with clavulanate (Figure 3E,F). These clones could not be expanded to the same extent as the clavulanate-responsive clones; thus more detailed assessments were not possible.

### Amoxicillin and Piperacillin-Responsive T-Cells Do Not Crossreact with Clavulanate and Tazobactam

**Figure 4:**
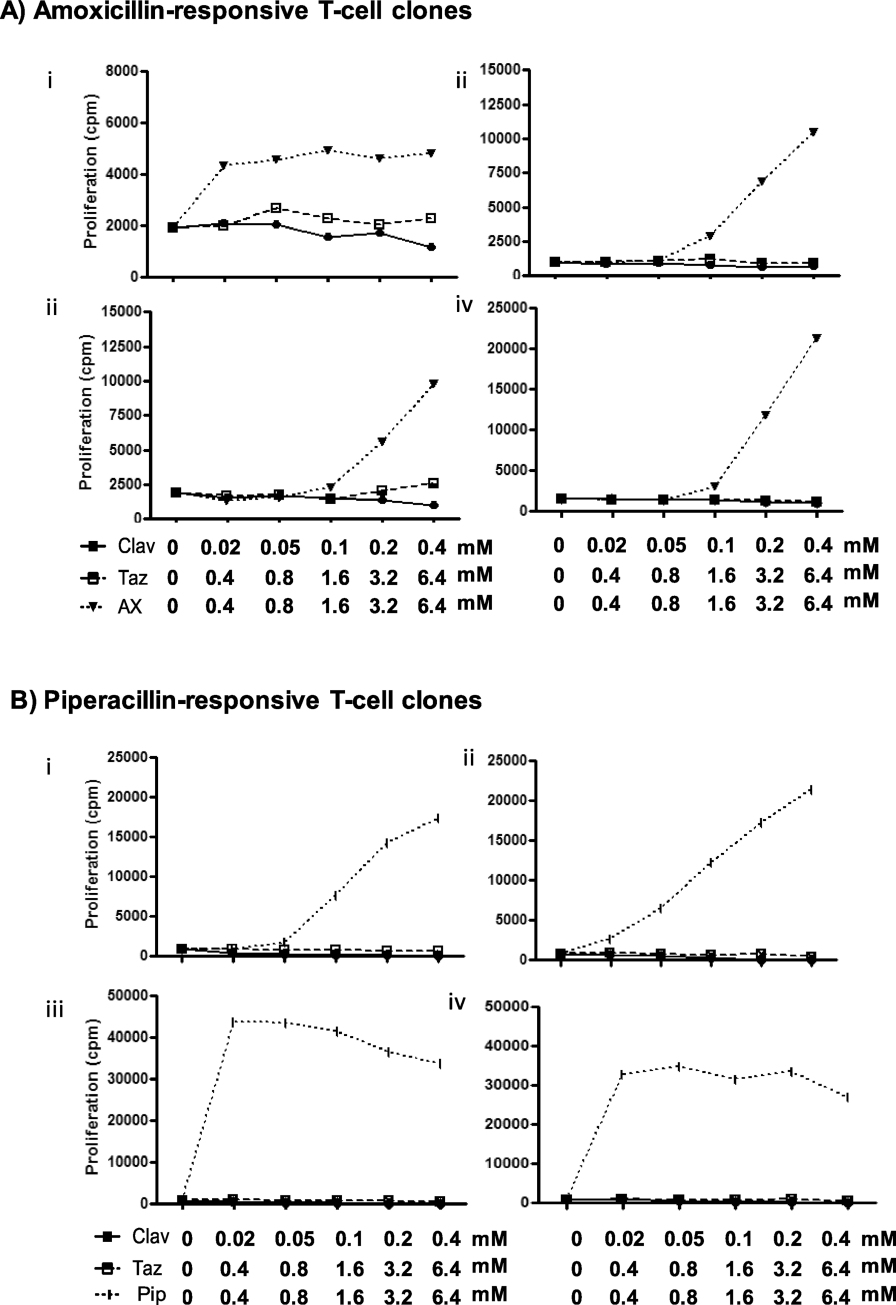
Crossreactivity of 6-lactam antibiotic-responsive T-cell clones with clavulanate or tazobactam. Proliferation assay showing that (A) amoxicillin- and (B) piperacillin-specific T-cell clones are not activated with either clavulanate or tazobactam. T-cell clones were cultured with APCs and either amoxicillin, clavulanate, tazobactam, or piperacillin for 2 days before analysis of proliferation by the addition of [^3^H]-thymidine. Results are expressed as mean counts per minute (cpm).

To further assess T-cell activity with clavulanate and tazobactam, the two drugs were cultured with APCs and T-cell clones responsive toward the structurally unrelated β-lactam antibiotics amoxicillin and piperacillin. Amoxicillin- and piperacillin-responsive clones proliferated in the presence of amoxicillin or piperacillin, respectively, but were not activated with clavulanate or tazobactam (Figure 4).

### T-Cell Clones Are Activated by Clavulanate and Tazobactam via a Processing-Dependent Pathway

**Figure 5:**
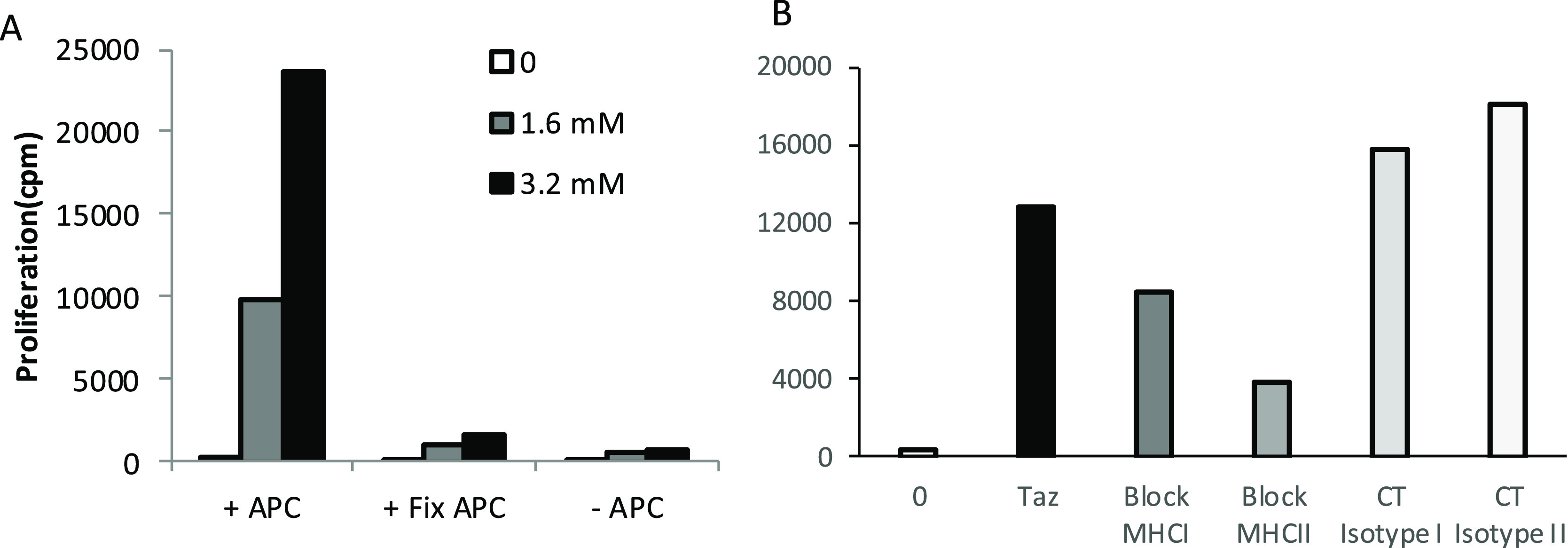
Tazobactam stimulates T-cell clones via an MHC-restricted and processing-dependent pathway. (A) T-cell clones were cocultured with drug and APC (+APC), glutaraldehyde-fixed APC (+Fix APC) or in the absence of APC (−APC) for 2 days before proliferation analysis by the addition of [^3^H]thymidine. (B) Proliferation assay to analyze tazobactam presentation to T-cell clones in the presence and absence of the MHC block. APC was incubated for 30 min at 37 °C with MHC class I and II blocking antibodies or isotype control before being cultured with drug and T-cell clones for 2 days. Proliferation was measured by the addition of [^3^H]thymidine. Results are expressed as counts per minute (cpm).

We have previously shown that CD4^+^ clones are activated with clavulanate via a pathway dependent on antigen processing.^19^ The current study focused on the pathway of CD4^+^ T-cell activation with tazobactam. Clones did not proliferate with tazobactam when APCs were omitted from the assay. Furthermore, fixation of APCs with glutaraldehyde, which inhibits antigen processing, reduced the strength of the induced tazobactam-specific response (Figure 5A). Pretreatment of APCs with MHC blocking antibodies before addition of tazobactam to the T-cell assay revealed that the CD4^+^ T-cell proliferative response was MHC class II restricted (Figure 5B).

### Characterization of Clavulanate and Tazobactam-HSA Adducts Formed In Vitro

**Figure 6:**
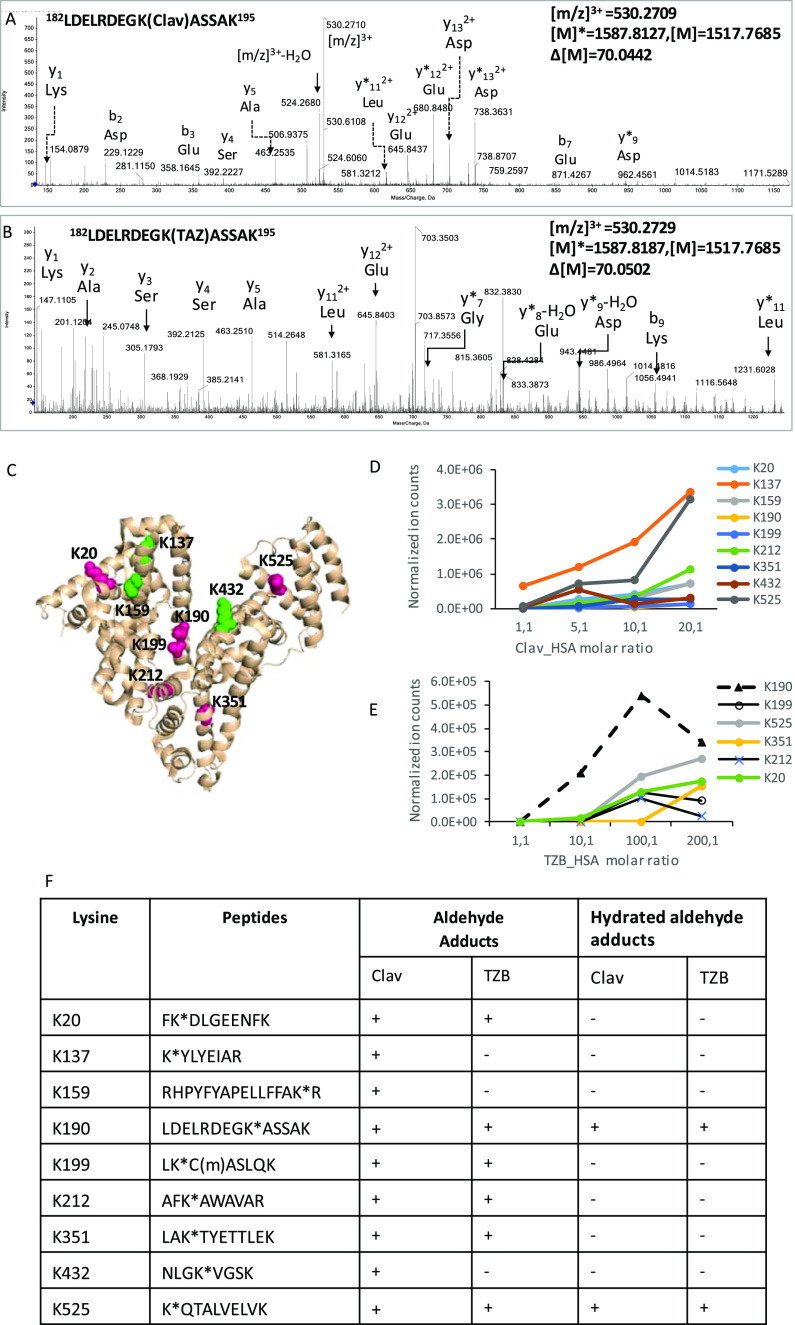
LC–MS/MS characterization of HSA adducts formed by tazobactam and clavulanate in vitro. Representative MS/MS spectrum of the albumin peptide 182LDELRDEGKASSAK195 modified at Lys190 with clavulanate (A). A similar adduct was also formed by tazobactam (B). The epitope map on HSA shows the lysine residues modified by tazobactam (at drug/protein molar ratio 100:1) or clavulanate (at drug/protein molar ratio 20:1); residues modified by both compounds are highlighted in pink and additional residues modified only by clavulanate are highlighted in green. Images are illustrated by PyMOL (The PyMOL Molecular Graphics System, Version 1.3 Schrödinger, LLC). The level of modification with clavulanate and tazobactam is concentration-dependent (D,E). (F) Summary of the HSA lysine residues targeted by clavulanate and tazobactam and the nature of the adducts formed.

**Figure 7:**
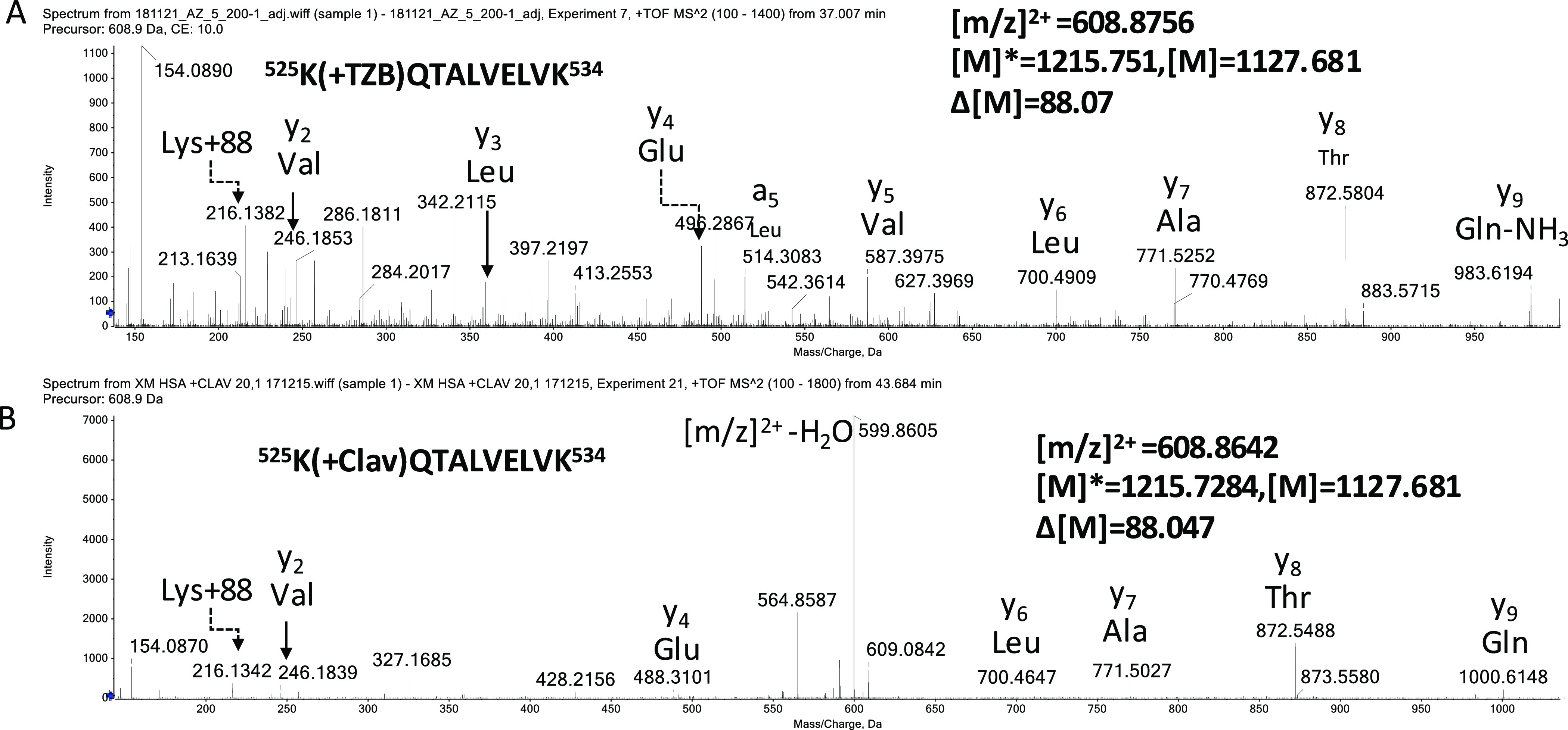
MS/MS spectra of tazobactam-modified peptides. A representative MS/MS spectrum shows a HSA peptide K*QTALVELVK that was modified by tazobactam with a mass addition of 88 Da (A). A similar adduct was also detected when clavulanate was incubated with HSA (B). Albumin peptides modified by clavulanate or tazobactam were identified in vitro. Clavulanate and tazobactam were incubated with HSA for 24 h at a drug protein molar ratio of 20:1 and 200:1, respectively.

**Scheme 1:**
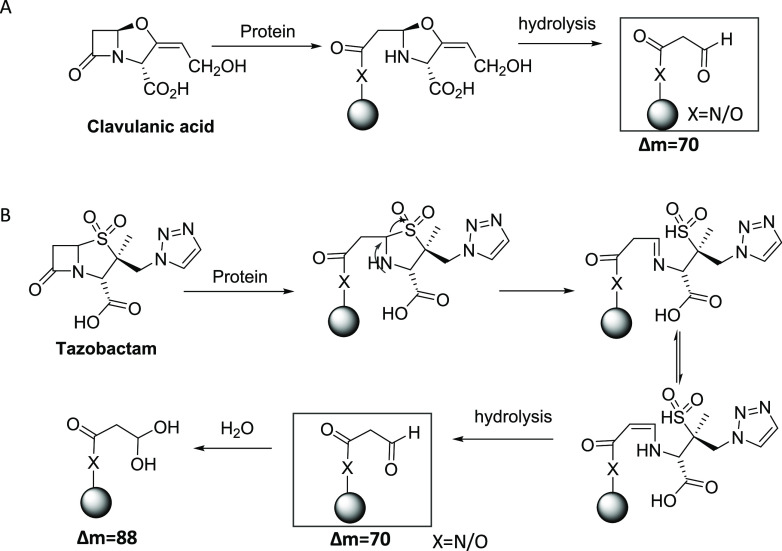
(A) Degradation of Covalently Bound Clavulanate to Lys19 and (B) Further hydrolysis Resulting in an Aldehyde/Hydrated Aldehyde Adduct

To characterize the putative protein adducts formed by clavulanate and tazobactam, HSA was incubated with a range of molar ratios of the drugs (1:1 to 200:1). As described previously, LC–MS/MS analysis of the tryptic digests of clavulanate HSA incubations revealed two types of adducts with a mass addition of 70 and 88 Da on proteins.^29^ The same adducts were detected when tazobactam was incubated with HSA. Figure 6A shows a representative MS/MS spectrum for a triply charged ion at *m*/*z* 530.27, corresponding to the tryptic peptide ^182^LDELRDEGKASSAK^195^ with an additional mass of 70 Da. The mass addition of 70 Da resulted from the degradation of covalently bound clavulanate to Lys190 (Figure 6A and Scheme 1A). The detection of the same adducts with exact the same mass following incubation of clavulanic acid and tazobactam with HSA suggested that tazobactam reacts with lysine residues through similar chemical mechanisms. A triply charged ion at *m*/*z* 530.27 corresponding to the tryptic peptide ^182^LDELRDEGKASSAK^195^ was detected (Figure 6B). The peptide sequence was confirmed by fragmentations that generated partial singly charged y and b series ions. The modification site was confirmed by y7* (*m*/*z* 718.36) and y11*(*m*/*z* 1231.60) with adduction of 70 Da. A total of seven lysine residues were found to be adducted with 70 Da when clavulanate was incubated with HSA at a molar ratio of 10:1, whereas six lysine residues were found to be modified by tazobactam when incubated with HSA at a molar ratio of 100:1 (Figure 6C). Lys190, Lys212, and Lys525 were modified by both drugs (Figure 6C, pink spheres). The +88 adducts were only detected on K190 and K525 for both compounds (Figure 7), which could be derived from hydration of the aldehyde.

The modification of HSA by tazobactam and clavulanate was found to be concentration-dependent. A semiquantitative analysis of modification at each site was performed by determining the area under the curve for the extracted masses of the modified peptides followed by normalization of the ion intensity against the total ion count for the sample. Notwithstanding the disparity in the ionization efficiency of the peptides, the relative abundance of clavulanate- and tazobactam-modified peptides increased with increased molar ratio of drug to protein, with Lys190 being preferentially targeted at low concentrations of tazobactam (Figure 6D,E). Figure 6F shows the lysine residues targeted by the two drugs and the nature of the adducts formed.

## Discussion

The hydrolysis of β-lactam antibiotics by bacterial β-lactamase is the major cause of bacterial resistance to this group of drugs. The combination of β-lactam antibiotics with β-lactamase inhibitors has significantly improved clinical efficacy. However, the addition of β-lactamase inhibitors may increase the risk of hypersensitivity reactions. For example, addition of clavulanic acid to amoxicillin has been shown to increase the risk of drug-induced liver injury.^30^ Many hypersensitivity reactions are mediated by the amoxicillin component of the drug combination; however, we have demonstrated that certain patients develop clavulanic acid-specific reactions.^12−14,19^ Clavulanic acid-hypersensitive subjects tolerate other β-lactam antibiotics, including amoxicillin,^17^ presumably because of the formation of distinct antigenic determinants. Very little is known about the cellular mechanisms of β-lactamase inhibitor hypersensitivity reactions and T-cell crossreactivity between different β-lactamases inhibitors. In this manuscript, we describe hapten-responsive T-cells from clavulanic acid and tazobactam hypersensitive patients that strongly crossreact. Importantly, mass spectrometric analysis also reveals that the two drugs form similar antigenic determinants by covalent modification of protein, which likely explains the T-cell crossreactivity.

Accurate diagnosis of patients with hypersensitivity reactions to β-lactamase inhibitors is difficult. This is because β-lactamase inhibitors are used in combination with β-lactam antibiotics, and they are not commonly assessed via skin testing or in vitro diagnostics as a single agent. Thus, it is challenging to differentiate whether hypersensitivity reactions are attributed to β-lactam antibiotics or β-lactamase inhibitors. Evaluation of the potential for crossreactivity of β-lactamase inhibitors is purely based on the chemical structure and known chemical reactivity, which sometimes can be misleading. The one exception is a recent study that evaluated immediate and nonimmediate piperacillin-tazobactam hypersensitivity from a large multicenter cohort.^23^ The skin-testing cross-sensitization pattern indicated that 3 of 87 patients may have developed selective reactions to tazobactam, and cross-reactivity was detected with clavulanic acid. In the current study, three clavulanic acid hypersensitive patients and one tazobactam hypersensitive patient were identified through diagnostic testing and PBMC were used to generate T-cell clones. CD4^+^ clones were found to proliferate and secrete IFN-γ in the presence of either clavulanate or tazobactam in a dose-dependent manner. CD8^+^ clones were not detected. The detailed analysis of crossreactivity between clavulanate and tazobactam showed that clavulanate-specific T-cell clones were activated with tazobactam and vice versa. The optimal concentration of clavulanate required to active the clavulanate-specific T-cell clones was 0.1–0.2 mM, while tazobactam-specific responses were detectable at higher concentrations. This is partly because tazobactam displays less intrinsic T-cell toxicity; however, the data do indicate that T-cells are more reactive toward lower clavulanate concentrations. Interestingly, T-cell activation was not observed when amoxicillin- or piperacillin-specific T-cell clones were tested against either clavulanate or tazobactam, suggesting that the antigenic determinants formed by clavulanate and tazobactam are different from those formed with amoxicillin or piperacillin.^12,29^

We have previously demonstrated that (i) clavulanate can generate distinct antigenic determinants that have little or no crossreactivity with those formed by amoxicillin^29^ and (ii) β-lactam antibiotics such as piperacillin form adducts on albumin that act as antigenic determinants for patient T-cells.^10,31^ Although albumin is unlikely to be the only immunogen generated in hypersensitive patients, it was selected as a surrogate protein to explore the binding of tazobactam and clavulanic acid. Nucleophilic attack of a lysine residue on the β-lactam ring of clavulanate generates an unstable adduct, which would generate multiple haptenic adducts following further decomposition. A total of seven types of clavulanate-HSA adducts were identified in in vitro incubations including adducts with mass addition of 70 and 88 Da. Furthermore, the +70 adducts were detected in amoxicillin-clavulanate exposed patients, indicating that these adducts could be potentially involved in the development of clavulanate hypersensitivity reactions. This chemical pathway for clavulanate protein-adduct formation was consistent with previous studies of clavulanate binding to the β-lactamase enzyme. Crystallographic studies revealed that a fragment of clavulanate covalently bound to the active site Ser70 of a class A serine β-lactamase, as either the cis or trans decarboxylated enamines.^32^ The adducts with mass addition of 70 and 88 Da were further confirmed by mass spectrometric analysis.^33^ Tazobactam, a triazolyl-substituted penicillanic acid sulfone β-lactamase inhibitor that is structurally distinct from clavulanate, has also been shown to inactivate the class A β-lactamases through acylation of the key serine residues on the enzymes.^34,35^ After acylation, tazobactam undergoes β-elimination, leading to the opening of the five-membered ring to form an imine. Further hydrolysis resulted in an aldehyde/hydrated aldehyde adduct (Scheme 1). Similar to the reaction with β-lactamases, the initial attack of a lysine residue on the β-lactam ring of tazobactam results in an unstable adduct, which undergoes further degradation and generates multiple adducts. Our mass spectrometric analysis revealed two adducts with a mass addition of 70 and 88 Da, which correspond to the aldehyde and hydrated aldehyde, respectively. Although tazobactam and clavulanate are structurally distinct, the chemical pathways of covalent binding to proteins are very similar, especially after the opening of the β-lactam ring. The formation of the same antigenic determinants is therefore likely responsible for the crossreactivity between clavulanate- and tazobactam-responsive T-cells.

Because clavulanate and tazobactam are able to covalently bind to proteins that require antigen processing and presentation to activate T-cells, it is not surprising that APCs were required for activation of T-cell clones with either clavulanate^19^ or tazobactam (Figure 5). Clavulanate- and tazobactam-responsive T-cell clones were activated with the drugs in the presence of irradiated APCs. In contrast, chemical fixation that blocks antigen processing or absence of APCs reduced the strength of the proliferation response, indicating that both clavulanate and tazobactam activate T-cells via a pathway dependent on protein processing.

In conclusion, our findings indicate that although clavulanate and tazobactam are structurally distinct, the antigenic determinants formed by both drugs are very similar, especially after the formation of the acylimine drug-protein complex. Although clavulanate appears to be more chemically reactive and able to stimulate T-cells at lower concentrations than tazobactam, the formation of the same antigenic determinants is likely responsible for T-cell crossreactivity observed between these β-lactamase inhibitors. These results can be used to produce better reagents for skin and in vitro diagnostic testing. In fact, a recent study has shown that synthetic antigenic determinants of clavulanic acid can induce improved responses in in vitro diagnostics.^36^

## Abbreviations

antigen presenting cells: APC
human serum albumin: HAS
stimulation index: SI
peripheral blood mononuclear cells: PBMC;
fluorescein isothiocyanate: FITC
phycoerythrin: PE
